# The practice of drug emergency supply in China during the COVID-19 pandemic: A policy mix perspective

**DOI:** 10.1371/journal.pone.0337700

**Published:** 2025-12-30

**Authors:** Zhang Jie-ying, Li Jun, Wang Xue-ya, Fan Rui-xue, Zhao Feng, Tang Yi, Zhang Jun-lin, Mao Ning-ying

**Affiliations:** International Pharmaceutical Business School of China Pharmaceutical University, Nanjing, Jiangsu, China; University of Maryland Baltimore, UNITED STATES OF AMERICA

## Abstract

**Background:**

Public health emergencies such as COVID-19 severely disrupt drug supply chains, creating sudden surges in demand, labor shortages, and regulatory challenges. Existing studies often focus on single supply chain stages, with limited systematic analysis of policy responses across the entire cycle.

**Objectives:**

This study aims to systematically analyze China's emergency drug supply policies by examining issuing agencies, developmental stages, policy tools, and supply chain links, to identify patterns, complementarities, and existing gaps.

**Methods:**

A total of 559 policy texts (110 national, 449 provincial) issued between December 2019 and February 2023 were collected. Content analysis and coding were conducted based on a four-dimensional framework. Social network analysis was applied to joint policy issuances to assess institutional collaboration and centrality.

**Results:**

National policies emphasized macro-level planning and dynamic adjustment: motivation and symbolic tools dominated in the burst stage, commands and regulations in the remission stage, and symbolic tools in later stages. Provincial policies relied more on rigid enforcement through commands and regulations, gradually shifting toward capacity-building in distribution and use. Finance, healthcare security, and regulatory agencies emerged as network hubs, while the distribution stage received the most policy support. Raw material supply was largely overlooked.

**Conclusions:**

National and provincial policies demonstrated complementary functions—national policies providing strategic direction and provincial policies ensuring operational enforcement. However, imbalances in policy tool use, weak shortage monitoring, and insufficient human resource policies remain. Future research should incorporate municipal-level measures, field-based evaluations, and international comparisons to optimize China's emergency drug supply governance.

## 1. Introduction

This study focuses on how governments can secure drug supply during public health emergencies, events characterized by sudden outbreak, rapid transmission, and high uncertainty. Such emergencies create exponential increases in demand, public panic, and regulatory failures [1]. After a public health emergency, the demand for and supply of drugs are in a complex state of imbalance [2]. On the one hand, social demand for medical supplies rises sharply in the short term, with certain resources being irreplaceable. As the emergency evolves, the focus of response shifts continuously, thereby increasing the complexity of supply needs. On the other hand, public health emergencies often damage human health, resulting in labor shortages across all links of the drug supply chain. According to estimates by the National Bureau of Economic Research (NBER), COVID-19–related health problems directly reduced U.S. labor force participation by about 0.2%, with average working hours still 5% lower a year later, and approximately 7% of workers absent for at least one week even after one year [3]. Meanwhile, due to the long R&D cycle, high difficulty, and high costs of drug production, there are significant delays in scaling up manufacturing capacity. In addition, the strict timeliness and conditions required for drug transportation further increase the difficulty of ensuring emergency supply. To solve this imbalance in a short period of time, it is insufficient to merely rely on market regulations. Instead, it requires direct government intervention [4]. However, given the staged and highly uncertain nature of public health emergencies, government policies must be flexibly adjusted as the situation evolves to reduce health risks. At the same time, the overlapping effects of multi-departmental policies increase the complexity of drug supply guarantees. the timeframe during which the government issues policies, the specific aspects it chooses to address, the compulsory nature of these policies, the methods of implementation, and particularly the combined effects of multiple policy tools, all contribute to a complex impact on the supply of drugs.

Various studies have investigated emergency drug supply policies. Existing research can be broadly divided into reserves, procurement, allocation, rationing and dispensing. In the area of reserves, Wang et al. analyzed China's policy documents related to reserve medical supply programs and summarized their major global forms [5]. Sun proposed a comprehensive evaluation system for emergency response capabilities based on analytical methods, analyzed emergency responses to public health emergencies and various functional management departments, and provided suggestions regarding emergency material reserve systems [1]. Lan et al. developed a model of government–enterprise joint stockpiling of essential medicines to mitigate shortages, proposed an option contract framework, and suggested that the government incentivize pharmaceutical firms through subsidies for Active Pharmaceutical Ingredient (API) reserves. Some studies have focused on the procurement stage [6]. As Arush Lal et al. reviewed policies in Latin America and the global health context, they examined strategies to secure essential medicine supply during the COVID-19 pandemic. The study emphasized that pooled procurement across countries and strategic partnerships are critical tools for leveraging economies of scale and ensuring affordable access to quality medicines [7]. Other studies have focused on the allocation of medical supplies, Yuan et al. constructed a tripartite evolutionary game model to study the allocation of medical supplies in public health emergencies under conditions of incomplete information. They found that the government should design a reasonable reward and punishment mechanism to ensure rational and orderly circulation of medical supplies, thereby reducing interference from government-owned nonprofit organizations (GNPOs) and hospitals [1]. Other research has focused on rationing and dispensing. For instance, Alpern et al. analyzed many states' adjustment measures for the one-month dispensing limit restriction beyond the COVID-19 pandemic, and proposed policy solutions to ensure uninterrupted access to medicines [8].

At the same time, some scholars have attempted to conduct systematic analyses of emergency drug supply policies. Sabine Vogler carried out a comprehensive study investigating how dozens of high-income countries—primarily in Europe, but also including Canada, Brazil, and Saudi Arabia—responded to the COVID-19-induced escalation of medicine shortages, mapping the range of government policy measures implemented as of 2023 [9]. Liu et al. developed an evaluation system for Public Health Emergency Response Policies (PHERPs) using content analysis of policy texts, expert consultations, and the PMC-Index model. They quantitatively analyzed 33 central-level PHERPs in China issued between 2003 and 2020, aiming to assess the formulation and implementation of these policies and identify areas for optimization [10]. Du and Lu (2023) focused on the dynamic evolution of China's governmental policy response to COVID-19 and constructed a three-dimensional framework of “policy subject–policy target–policy instrument.” Through quantitative analysis of policy texts and social network analysis covering the three-month period from January 20 to March 26, 2020, their study revealed shifts in the internal focus of the policy mix and the patterns of instrument combinations at different stages of the pandemic. These findings contribute to understanding how the policy mix adjusts with the evolving situation to enhance effectiveness [11].

Overall, existing studies mostly focus on a specific stage of the emergency drug supply chain, while systematic analysis of policies as a whole remains insufficient. As the most severe public health emergency in recent years, the COVID-19 pandemic has provided a wealth of practical cases for relevant research. China introduced a large number of emergency policies during the pandemic, but there is still a lack of systematic analysis of these policies over the complete cycle. Therefore, this study constructed a four-dimensional analysis framework of the issuing agency, developmental stages, policy tools, and drug supply chain to systematically analyze the issuing of policies in China during public health emergencies, examining how policies are combined in terms of who formulates them, when they are issued, how they are implemented, and which stage of the supply chain they targe, and to identify existing problems that require urgent attention. Finally, suggestions on how to scientifically create policies under emergencies for public health in the long run are provided.

## 2. Methods

### 2.1. Construction of the four-dimensional analysis framework for policies of drug supply under emergency

**Fig 1:**
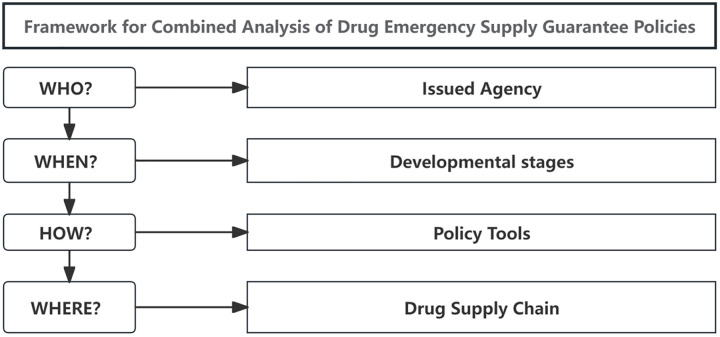
Four-dimensional analysis framework of Issuing Agency - Issuing Period - Policy Tools - Drug Supply Chain.

The drug availability fluctuates depending on the developmental stage of public health emergencies. Drug supply support is strongly linked to drug research and development (R&D), manufacturing, distribution, use, and other processes. Different supply chain processes play specific roles in various developmental stages of an emergency. An appropriate selection and combination of policy tools are necessary to achieve an orderly supply of medicines. Therefore, the overall structure of policy tools must be assessed. It is only possible to guarantee a timely, accurate, orderly, and safe supply of medicines by carefully choosing appropriate policy tools for various processes in the drug supply chain and developmental stages of the emergency. As a result, by comprehensively considering the complexity of drug supply and the multi-stage characteristics of public health emergencies, the team, based on existing research and practical needs, builds a four-dimensional analysis framework, consisting of: “Who issues the policies (Issued Agency), When the policies are introduced (Developmental Stages), How the policies take effect (Policy Tools), and Where the policies are applied (Drug Supply Chain).”. We then used this four-dimensional framework to analyze the policies of the nation and representative provinces regarding drug supply during emergencies (Fig 1).

#### 2.1.1. Issuing agency.

In this study, we chose relevant departments related to drug supply under emergency conditions as the criteria for the dimension of the issuing agency, including the government, health security administration, health commission, administration for market regulation, drug administration, public finance department, scientific and technological commission, transportation department, development and reform commission, taxation administration, and administration of customs.

#### 2.1.2. Developmental stages.

**Fig 2:**
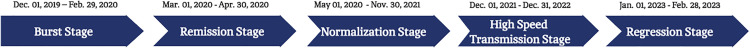
Development stages of public health emergencies.

Public health emergencies are divided into four stages from the beginning to end—the outbreak, de-escalation, normalization, and the fallback periods—representing the process from the outbreak of public health emergencies, dramatic increase in the number of infected people, stabilization of the affected area, and the final gradual recovery, respectively [12]. Public health emergencies are divided into five developmental stages in this study based on the number of infections, deaths, and related policy adjustments in China during each period from December 2019 to February 2023 [13], as well as the division of COVID-19 developmental stage in the relevant literature and the statistical report of the Chinese Center for Disease Control and Prevention. The five developmental stages in this study are: (1) the burst stage (December 01, 2019–February 29, 2020); (2) remission stage (March 01, 2020–April 30, 2020); (3) normalization stage (May 01, 2020–November 30, 2021); (4) transmission stage (December 01, 2021–December 31, 2022); and (5) regression stage (January 01, 2023–February 28, 2023) (Fig 2).

#### 2.1.3. Policy tools.

This study refers to Schneider and Ingram's [14] and McDonnell and Elmore's [15] classification theory of policy tools, as well as existing research results on the evaluation of drug supply and public health emergency policies, combined with the actual work of drug supply under public health emergencies. The basic tools of the drug supply policy in public health emergencies are divided into four categories: commands and regulations, capacity-building, motivation, and symbolic and hortatory tools. The specific meanings and distinctions of each policy tool are listed in Table 1.

**Table 1:** Policy tool meanings and distinctions.

Tool	Meaning	Element	Object	Form	Example
Commands and regulations	When a public health emergency occurs, the government or policy-making department uses public power and political authority to compulsorily regulate and restrain relevant parties, and stipulates or prohibits that the target group take intended action.	Rule	Encourage compliance	Quality supervision	Strengthen the supervision and inspection of pharmaceutical and medical device manufacturers’ compliance with Good Manufacturing Practice (GMP) standards.
				Price monitoring	Business operators shall not fabricate or spread information about price increases of epidemic prevention supplies and essential goods.
				Shortage monitoring	Procurement platforms shall strengthen monitoring of procurement and supply, incorporating all pandemic-related medicines and medical consumables into emergency monitoring systems.
				Code of Conduct	prohibiting the release of illegal advertisements containing claims such as “prevention, treatment, or cure of COVID-19,”
Capacity-building	Developing information technology and science and technology through capital investment, technical support, and human resources investment to ensure the normal supply of drugs in cases of public health emergencies.	Investment	Enhance ability	Platform construction	Improve and enhance integrated information platforms for the allocation of protective materials and the supply and demand of medical services; based on geospatial information, dynamically manage key resources within the province—including epidemic control and treatment institutions, personnel, vaccines, equipment, and supplies—and ensure their visualized presentation.
				Capital investment	Supporting enterprises by insuring advance payments for imported epidemic prevention materials
				Human Resources	Dynamic monitoring of employment should be conducted for enterprises producing epidemic prevention and control materials before and after their resumption of work. Meanwhile, careful analysis should be made of the labor shortages of enterprises that have already resumed work, predictions should be made on the labor demand of enterprises after their full resumption of work, and preparations for responses should be made in advance.
				Technical guidance	Deploying regulatory and technical staff across districts to provide on-site services, guidance, and supervision to ensure medical device manufacturers increase production capacity.
Motivation	Use policy preferences, punishment, and other positive or negative inducements to encourage and guide target groups to implement the expected values and actions, to realize the normal supply of drugs in public health emergencies.	Encouragement	Guide behavior	Fast track	Opening a fast track to accelerate the review and approval of registration applications for urgently needed pharmaceutical products during the pandemic
				Regulatory relaxation	Simplifying customs clearance procedures for imported medical devices
				Financial incentives	For enterprises producing key epidemic prevention and control materials, newly purchased equipment for capacity expansion shall be allowed to be deducted in full as current expenses before enterprise income tax.
Symbolic and hortatory	Implement value guidance for relevant parties, change the policy preference perception of target groups, and lead social identity, to achieve the normal supply goal of drugs in public health emergencies.	Calling	Form values	Public opinion and publicity	Strengthen news and publicity efforts, proactively release information, and provide positive guidance on the production and allocation of key prevention and control materials as well as on ensuring the stable supply and prices of daily necessities.
				Social mobilization	Social relief organizations such as the Red Cross Society and charity federations shall strictly carry out donation and relief activities in accordance with the law, and relevant information shall be disclosed in a timely manner.

#### 2.1.4. Drug supply chain.

The drug supply chain refers to the network chain organization jointly established by upstream and downstream enterprises in the process of drug production and distribution. It involves multiple processes, including drug R&D, raw material supply, manufacturing, distribution, and use [16]. The term “whole chain” is used to indicate that the policy text does not explicitly specify which particular part of the drug supply chain it refers to. Instead, it encompasses the entire supply chain process without detailing specific segments, for example, “emergency review and approval” is not clearly defined as the “review and approval” in the R&D, manufacture, or distribute processes; therefore, such circumstances correspond to the entire chain process.

### 2.2. Text retrieval and classification of policies about drug supply under emergency

#### 2.2.1. Selection of research areas.

Firstly, relevant policy documents issued at the national level are included in the scope of this study. Secondly, representative provinces are selected by comprehensively considering geographical regions and infection data. According to comprehensive geographical standards, China's territory is divided into seven major regions: Northeast China, East China, North China, South China, Central China, Southwest China, and Northwest China [17]. The total number of infections and the infection rate in each provincial region from December 2022 to January 2023 are counted [18]. Then, the provincial regions with higher infection data in each of the seven regions are determined respectively, and 11 provincial regions including Jilin, Shanghai, Jiangsu, Zhejiang, Beijing, Hebei, Tianjin, Hubei, Guangdong, Chongqing, and Shaanxi are selected.

#### 2.2.2. Retrieval of policy texts.

Using “epidemic situation (疫情)”, “public health emergencies (突发公共卫生事件)”, “drug supply guarantee (药品供应保障)”, “drug emergency supply (药品应急供应)”, and “drug emergency guarantee (药品应急保障)” as keywords, a full-text search is conducted through government official websites. Laws, administrative regulations, departmental rules, and local regulations issued on the official websites of the People’s Government, the Medical Security Bureau, the National Health Commission, the Market Supervision Administration, the National Medical Products Administration, financial departments, science and technology departments, transportation departments, the National Development and Reform Commission, the Tax Bureau, the Customs, etc. at the national and provincial levels from December 1, 2019, to February 28, 2023, are collected. And the Pkulaw legal database is used to check for any missing policies among the collected ones.

The “drugs” in this study mainly include drugs or medical devices used for epidemic prevention, control, or treatment (including medical masks, nucleic acid test reagents, antigen test reagents, etc.). The inclusion criteria for the policies are as follows: (1) Policy documents that clearly mention the drug supply guarantee under the COVID-19 epidemic in the title or the main body content; (2) The main types of policies selected include laws, notices, opinions, reports, suggestions, regulations, provisions, detailed rules, and plans, etc.; (3) For policy documents that only mention “drug supply guarantee under the COVID-19 epidemic” and the like in only one chapter or a few sentences, only the mentioned parts are selected for coding and analysis.

**Fig 3:**
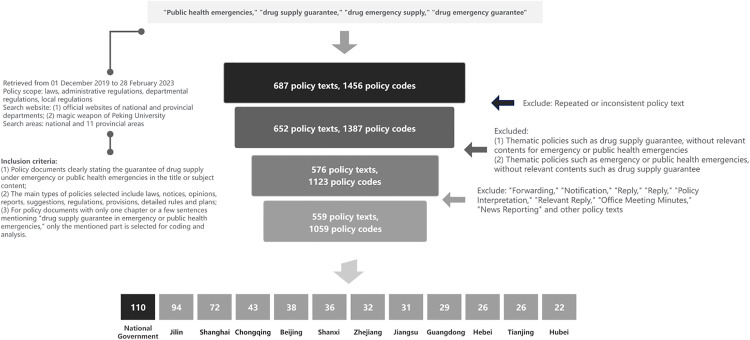
Policy text retrieval and screening process.

The exclusion criteria for the policies are as follows: (1) Policy texts that are repetitive or do not conform to the content requirements; (2) Policies with the theme of drug supply guarantee, etc., without relevant content targeting the COVID-19 epidemic; (3) Policies with the theme of COVID-19 epidemic prevention and control, etc., without relevant content targeting drug supply guarantee; (4) “Forwards”, “Notifications”, “Approvals”, “Replies”, “Policy Interpretations”, “Related Reply Letters”, “Meeting Minutes of Office Meetings”, “News Reports”, etc. (Fig 3).

To minimize bias during this process, six researchers independently participated in the retrieval and preliminary screening of documents. Each policy text was reviewed by at least two members, and any disagreements about inclusion or exclusion were discussed within the group until consensus was reached.

### 2.3. Policy text coding and classifications

Excel 2019 software was used to code policy-related content. National-level policy text was coded as “A – National level policy number section-specific clause (A-国家级政策编号-章节-具体条款),” and provincial policy text was coded as “B – Provincial policy number section-specific clause (B-省级政策编号-章节-具体条款).” According to the specific content encoded in policy documents, a coding manual was developed based on the framework: each policy document was annotated with its issuing body under the “issuing agency” dimension; the timing of issuance was mapped to the pandemic “developmental stage”, the measures adopted were categorized into one of the four “policy tools” types (commands and regulations, capacity-building, motivation, or symbolic and hortatory), and the targeted process was coded under the “drug supply chain” dimension (R&D, manufacturing, distribution, use, or whole chain). As a result, each policy was transformed into a set of four-dimensional labels.developmental stages (The example is shown in Table 2). To further reduce bias, the coding work was also conducted by six researchers. Each policy item was independently coded by two members, and discrepancies were resolved through group discussion until consensus was reached.

**Table 2:** Examples of policy coding.

发文时间 (Issuance Date)	发文单位 (Issuing Agency)	政策编号 (Policy No.)	政策名称 (Policy Title)	内容分析单元 (Content Analysis Unit)	编码号 (Code No.)	隶属节点 (Classification Node)			
						政策工具 (Policy Tool)	内容 (Content)	药品供应链 (Drug Supply Chain)	发展阶段 (Developmental Stage)
2020/1/22	市场监管总局 (State Administration for Market Regulation)	1	《药品注册管理办法》 国家市场监督管理总局令第27号 (Drug Registration Administration Measures, SAMR Order No.27)	药物临床试验期间, 符合以下情形的药品, 可以申请附条件批准: (三) 应对重大突发公共卫生事件急需的疫苗或者国家卫生健康委员会认定急需的其他疫苗, 经评估获益大于风险的. (During clinical trials, drugs meeting the following conditions may apply for conditional approval: (3) vaccines urgently needed for major public health emergencies or other urgently needed vaccines recognized by the National Health Commission, where benefits outweigh risks after evaluation.)	A-1-4-2-63	激励 (Motivation)	绿色通道 (Fast track)	药品研发 (R&D)	爆发期 (Burst Stage)
2020/3/27	北京市药品监督管理局 (Beijing Medical Products Administration)	2	北京市药品监督管理局关于做好疫情防控支持医药企业复工复产的通知 (京药监发 [2020] 78号) (Notice of Beijing MPA on Supporting Pharmaceutical Enterprises in Resuming Work and Production for Epidemic Prevention and Control, 2020 No.78)	(三) 加快防疫用医疗器械审批备案. 在疫情防控过程中, 开通绿色通道, 制定了《关于对防控疫情用第二类医疗器械实行应急审批的通知》, 启动联动机制, 快速审批等方式, 对疫情防控有关产品实施应急审批, 加快产品上市. (Accelerate the approval and filing of medical devices for epidemic prevention. During epidemic prevention and control, open a fast track, issue the “Notice on Emergency Approval for Class II Medical Devices for Epidemic Prevention,” launch a joint mechanism, and implement rapid approval to expedite product launch.)	B-1-1-2-3	激励 (Motivation)	绿色通道 (Fast track)	药品生产 (Manufacture)	缓和期 (Remission Stage)
2021/7/28	河北省人民政府办公厅 (General Office of Hebei Provincial People’s Government)	3	河北省人民政府办公厅印发关于全面加强药品监管能力建设若干措施的通知 (冀政办字 [2021] 95号) (Notice on Several Measures to Comprehensively Strengthen Drug Regulatory Capacity, Hebei Gov. Office Doc. 2021 No.95)	(十二) 完善应急管理体系机制. 各级政府要制定完善药品 (疫苗) 安全突发事件应急预案, 健全应急管理机制. 强化应对突发重大公共卫生事件中检验检测, 体系核查, 审评审批, 监测评价等工作的统一指挥与协调. 加强全省药品安全应急体系建设, 强化应急能力培训, 采取多种形式组织常态化药品安全应急演练, 提高各级负责药品监管机构的应急处置能力. 加强应急检验设施设备维护, 强化应急检验队伍培养和关键技术研究. (Improve the emergency management system. All levels of government should formulate and improve contingency plans for drug (vaccine) safety incidents, strengthen the emergency management mechanism, and unify command and coordination of testing, inspection, review, approval, and evaluation during major public health emergencies. Enhance the provincial drug safety emergency system, strengthen emergency training, conduct routine emergency drills, and improve the response capacity of regulatory agencies. Strengthen maintenance of emergency testing facilities and equipment, and enhance the training of emergency teams and research on key technologies.)	B-2-1-2-12	命令与规制, 象征与劝诫 (Commands and Regulations; Symbolic & hortatory)	行为准则、社会动员 (Code of Conduct; Social mobilization)	药品生产 (Manufacture)	常态化时期 (Normalization Stage)
...	...	...	...	...	...	...	...	...	...

### 2.4. Combined analyses of policies about drug emergency supply

After two rounds of reviews and screening of the policy text content, 559 qualified policies and 1,059 policy codes were obtained. The policies issued by China on drug supply under the pandemic involved multiple processes and stakeholders in the drug supply chain. Policy content focused on the characteristics of different drug supply processes and the needs of various stakeholders. There were differences in the number of policies released over time. The combined analysis of policies regarding drug emergency supply was conducted by integrating multiple dimensions, including issuing agencies, public health emergency development stages, policy tools, and the drug supply chain. The focus was on understanding how policies were issued by various agencies/units and how these policies evolved across different stages of the pandemic. The analysis also examined the types of policy tools used, such as symbolic, hortatory, command, regulation, capacity-building, and motivation tools, and their application at both the national and provincial levels.

To ensure clarity in the methodological approach, our analysis was primarily descriptive. We first conducted counts and proportions of policies across the four analytical dimensions (issuing agency, developmental stages, policy tools, and drug supply chain). In addition, we constructed a co-issuance network of agencies and applied centrality measures to analyze the relative positions and influence of different institutions in policy collaboration. These descriptive statistics were used to identify the distributional characteristics of policies in each dimension. In addition, we performed cross-tabulation analyses to examine patterns across two or more dimensions (policy tools × developmental stage, policy tools × drug supply chain, policy tools × developmental stage × drug supply chain). This allowed us to explore how different policy tools were combined, how issuing authorities coordinated their actions at different pandemic stages, and whether particular tools were disproportionately applied to certain supply chain stages.

The data used in this study were obtained from publicly available databases and do not involve personal privacy or sensitive information.

## 3. Results

After two rounds of text search, 119 policy texts related to drug supply guarantees at the national level and 568 policy texts at the provincial level under public health emergencies were obtained through preliminary screening, totaling 687 policy texts as our study subjects. For the policy texts obtained from the primary screening, a total of 559 final screening policies were obtained, including 110 at the national level and 449 at the provincial level, excluding repeated policy texts and those that only mentioned “emergency situations” without clearly indicating COVID-19 and emerging keywords without relevant substantial information.

### 3.1. Single-dimensional analysis: Issuing agency

Through the analysis of all policy texts included in the study, it was found that many subjects issued documents for relevant policies. In this study, 15 subjects with many documents issued were selected for feature analysis based on the number structure of documents issued. In terms of the total number of documents issued, the units that issued more documents were often the general office of state council/local government, public finance department, Drug Administration, Health Security Administration, Administration for Market Regulation, Health Commission, Development and Reform Commission, and Transportation Department, which accounted for approximately 89.45% of the total (Table 3). The number of documents issued jointly and those issued separately for statistical policies showed that drug supply guarantee policies in China were predominantly issued separately, 66.55% of the time. The number of joint documents issued by multiple departments was less than 30% (Table 4). Overall, the synergy between our policy subjects is not high, and it is necessary to strengthen the coordination and cooperation between various policy-making bodies.

**Table 3:** List of policy-issuing agencies.

Policy-issuing agencies	Number of documents issued	Number of documents issued individually
General Office of State Council/Local Government	101	98
Public Finance Department	85	31
Drug Administration	72	55
Health Security Administration	68	43
Administration for Market Regulation	66	51
Health Commission	52	15
Development And Reform Commission	33	11
Transportation Department	23	20
Scientific and Technological Commission	21	18
Taxation Administration	15	5
Ministry of Industry and Information Technology	14	4
Banking and Insurance Regulatory Commission	13	9
Administration of Customs	8	2
Economic and Information Technology Department	8	7
Ministry of Civil Affairs	6	3

**Table 4:** Situation of policy making bodies issuing policies jointly.

Issuing agency	Number of documents issued			Percentage of the number of documents issued to the overall number (%)
	National level	Provincial level	Total	
Two issuing bodies	5	27	32	5.72%
Three or more issuing bodies	15	35	50	8.94%

**Fig 4:**
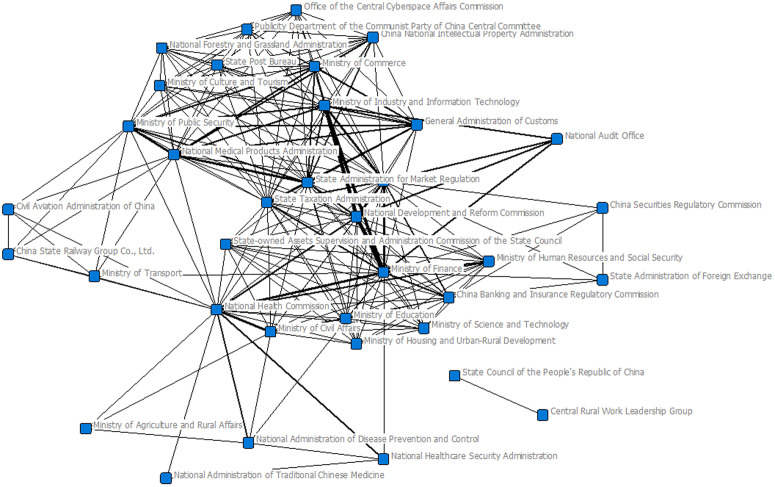
Social network of jointly issued policies at the national level.

From the joint issuance network and the centrality indicators at the national level (The centrality results of the national-level joint policy-issuing agency network are shown in S1 Table), it can be observed that economic and financial departments such as the Ministry of Finance (Degree = 38, Closeness = 54), the National Development and Reform Commission (Degree = 33, Closeness = 56), and the People's Bank of China (Degree = 37, Closeness = 48) occupy core positions in the network, with high degree centrality and closeness centrality. This indicates that their joint issuance relationships with other agencies are the most extensive, allowing them to quickly reach the entire network. Meanwhile, the National Health Commission (betweenness = 98.2120) shows the highest betweenness centrality, highlighting its role as a crucial bridge in cross-departmental coordination. By contrast, the eigenvector centrality of most agencies remains low, suggesting a clear core–periphery structure in joint policy issuance. Overall, the policy issuance network presents a “dual-core structure” characterized by “economic and financial dominance with health as the hub,” reflecting both the leading role of macro-regulatory agencies and the essential bridging function of public health authorities in emergency policy-making (Fig 4).

**Fig 5:**
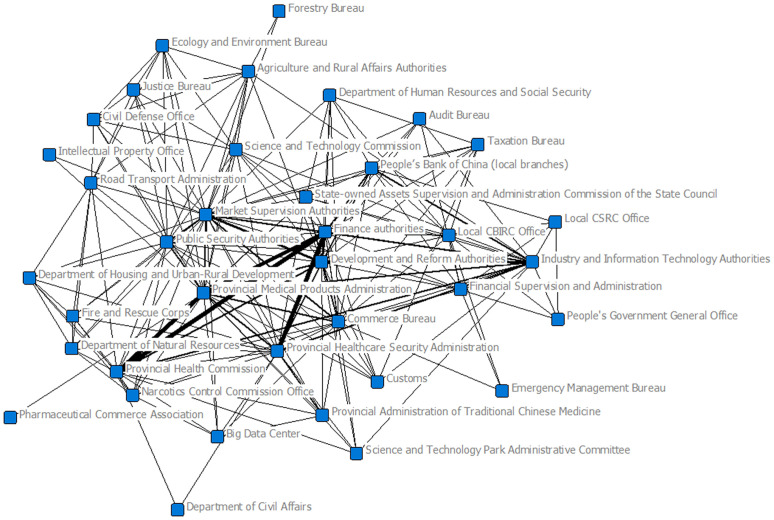
Social network of jointly issued policies at the provincial level.

From the provincial-level joint issuance network and centrality indicators (The centrality results of the provincial-level joint policy-issuing agency network are provided in S2 Table), Finance authorities hold the highest degree centrality (Degree = 140), indicating the broadest joint issuance relationships and a core position in the network. Development and Reform authorities (Degree = 104) and Market Supervision authorities (Degree = 108) also exhibit high degree values, reflecting their active involvement in joint policy issuance. Regarding closeness centrality, the General Office of the Provincial Government (Closeness = 131.0) ranks highest, suggesting strong accessibility to the entire network and the ability to quickly disseminate or gather information. Departments such as the Emergency Management Bureau (Closeness = 87.0) and the Department of Civil Affairs (Closeness = 104.5) also show relatively high closeness, underscoring their coordination roles in public health emergencies. For eigenvector centrality, the Provincial Healthcare Security Administration (Eigenvector = 0.9550), Finance authorities (Eigenvector = 0.8395), and the Provincial Medical Products Administration (Eigenvector = 0.5465) score the highest, suggesting that these agencies are not only highly active but also well connected with other influential actors, serving as authoritative nodes in the provincial policy network. In terms of betweenness centrality, the Market Supervision authorities (Betweenness = 353.8) and the Provincial Medical Products Administration (Betweenness = 137.8) stand out, highlighting their function as critical “bridges” in cross-sector coordination, facilitating the flow of information and policy resources across the network. Overall, the provincial joint issuance network demonstrates a structure of “finance and economic departments as leaders, healthcare security and medical products administrations as core hubs, and market supervision as a key bridge.” This reflects the leading role of macroeconomic agencies in policy integration while emphasizing the pivotal functions of healthcare security and regulatory authorities in safeguarding drug supply policies (Fig 5).

### 3.2. Single-dimensional analysis: Development stages

**Fig 6:**
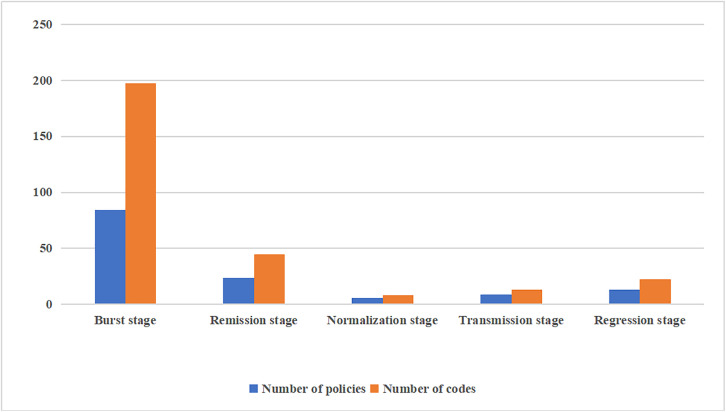
Policies and code quantities at each stage of national and provincial COVID-19 development/month.

The number of policies experienced a surge in the outbreak period, followed by a rapid decrease, and subsequently a gradual increase through each developmental stage of the pandemic (Fig 6). Owing to the different durations of each developmental stage, the number of policies were affected by time-span. This study presents a trend diagram of the number of policies issued at each stage in the following calculation mode: the number of policies at each stage of the pandemic's development (i.e., the number of policy codes) divided by the month that this stage lasted for. Overall, the fluctuations in both the number of policies and the number of policy codes were substantial across different stages (variance of 1063.56 and standard deviation of 32.61 for policies; variance of 6349.45 and standard deviation of 79.68 for codes), indicating significant stage-specific differences in policy supply during the pandemic. The number of policy releases during the development stage of the pandemic surged during the outbreak period, first presenting the characteristics of a rapid decline (a sharp decrease of 71.54% from the outbreak stage to the remission stage, and a further 74.79% decrease from the remission stage to the normalization stage), followed by a slow recovery. From the normalization stage to the transmission stage, the number of policies and policy codes increased by 48.76% and 63.95%, respectively, suggesting a rebound of policy support as the pandemic entered the transmission stage. By the regression stage, this upward trend continued, with policy numbers rising by 44.44% and codes by 67.16%. Policy-making agencies in China have issued relevant policies to guide and improve drug support at various stages of the pandemic.

### 3.3. Single-dimensional analysis: Policy tools

According to statistics from the coding results, the balance between the internal use capacity-building and commands and regulations tools must be improved. As per the proportion of the four types of policy tools, symbolic and hortatory tools are most preferred at the national level, but are used less frequently in provinces. National policymakers tend to guide relevant parties in values and lead social identities in the form of calls to achieve their goals, so that provinces issue more operable policies in response to national calls.

At both the national and provincial levels, policymakers in China tend to use motivation tools, of which the fast track is most widely used in overall secondary policy tools, although it has rarely played a role in guaranteeing drug supply during the pandemic through more direct investment in platforms, technologies, manpower, and funds. Judging from the secondary policy tools of policy tools compared to the provincial level, national policymakers adopt more means of social mobilization and call on the whole society to guarantee the supply of drugs while paying more attention to the quality of drugs under strict supervision.

**Fig 7:**
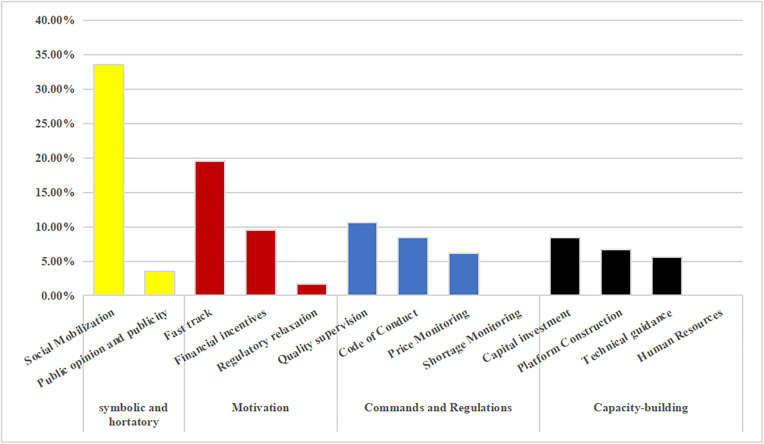
Percentage of classified use for policy tool types at the national level.

**Fig 8:**
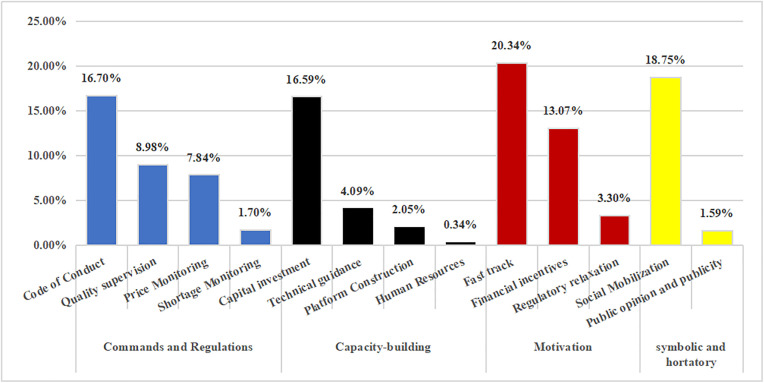
Percentage of classified use for policy tool types at the provincial level.

In addition, national and provincial policymakers favor a fast track, code of conduct, capital investment, and financial incentive policy tools. However, there are a few concerns regarding human resource and monitoring shortages. In addition, the internal use of capacity building, command, and regulation tools must be balanced when policy-making agencies formulate policies (Figs 7 and 8).

### 3.4. Single-dimensional analysis: Drug supply chain

**Fig 9:**
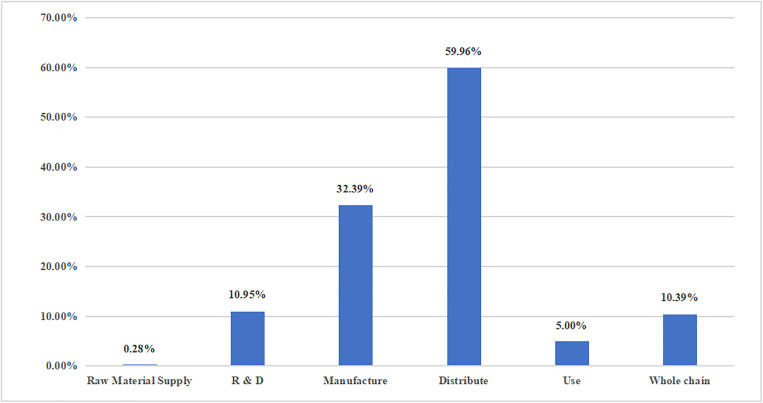
Distribution of national and provincial drug supply chain dimensions.

The policies regarding drug supply issued by the state during the pandemic intervened in different processes of the drug supply chain, paying more attention to drug distribution and less attention to raw material supply. The highest frequency of use was in the circulation of drugs, with a frequency and proportion of 635 times (59.96%), indicating that the state attaches significant importance to drug distribution. In order to ensure drug supply, multiple measures are taken to organize the source of goods and ensure the smooth transportation of drug logistics; the policy role of manufacturing and drug R&D is generally maintained at a relatively high level, with a frequency and proportion of 343 times (32.39%) and 116 times (10.95%) (Fig 9), respectively, indicating that the state is strengthening the R&D of drugs for pandemic prevention and control, expecting to provide strong scientific and technological support for overcoming the pandemic, while guiding pandemic prevention and control enterprises to return to work and production, accelerating manufacturing progress and attempting to meet the needs of pandemic prevention and control treatment on the basis of ensuring drug safety and effectiveness; after the pandemic occurred, enterprises had to dispatch raw materials to intensify production; however, China has less than 1% of policies for raw material supply, with much needed attention in this regard.

### 3.5. Multidimensional integrated analysis of policy tools and the drug supply chain

**Fig 10:**
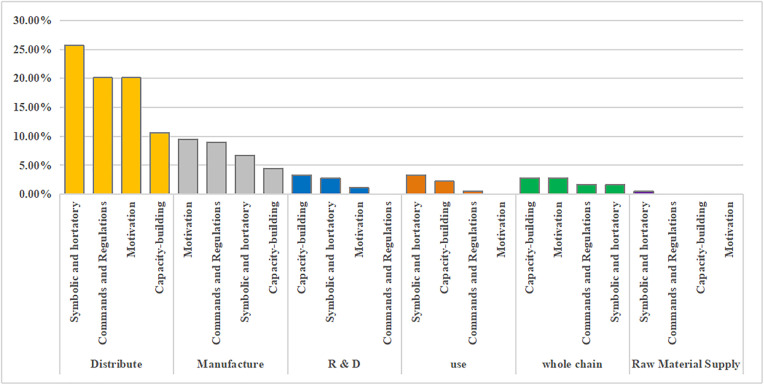
Percentage of policy tools used in the national drug supply chain.

**Fig 11:**
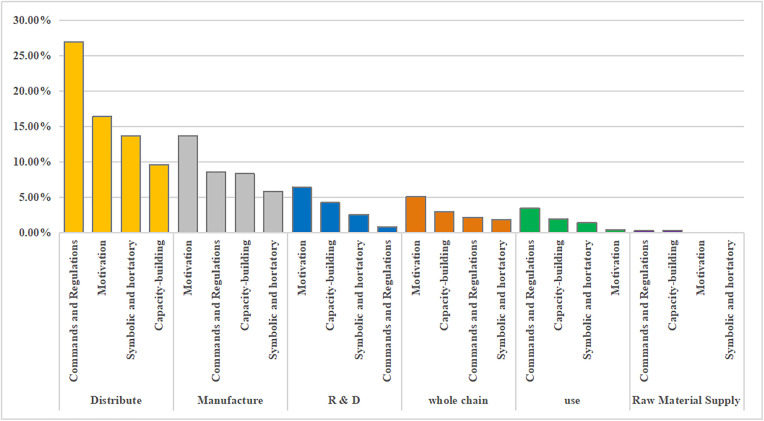
Percentage of policy tools used in the provincial drug supply chain.

By comparison, both national and provincial policies focus on the distribution stage, dominated by commands and regulations and motivation tools. In terms of R&D support, national authorities tended to adopt more direct capacity-building tools (3.35%) to enhance the overall drug R&D capacity, while all provincial regions paid more attention to adding fast tracks to the process of drug R&D review and approval, adopting financial incentives to accelerate the speed of R&D review and approval, and enhance R&D motivation of relevant organizations. However, the use of motivation tools by national policy-making agencies is relatively lacking, accounting for only 1.12% of all policies. In the manufacturing stage, national and provincial policy-making institutions tended to use motivation policy tools to accelerate the review and approval of enterprise expansion, solve enterprises' worries by promising to recover the remaining products, and take relevant financial incentive measures to enhance drug manufacturers' motivation to return to work, production, and expanding production lines, but provincial policies employed capacity-building tools more frequently than national ones (8.30% vs. 4.47%). During the process of drug distribution and use, China's national policy-formulating institutions tend to use relatively mild symbolic and hortatory tools, functioning as macro-level guidance and order coordination, whereas provincial policy-formulating institutions use more commands and regulations tools, undertaking concrete law enforcement and ensuring policy implementation (Figs 10, 11).

### 3.6. Multidimensional integrated analysis of policy tools and developmental stages

In comparison, central and provincial policies reveal a distinct hierarchical division of labor and strategic divergence in the dynamic adjustment of policy tools. During the burst stage, the central government emphasized nationwide strategic mobilization, primarily adopting motivation tools (36.46%) and symbolic and hortatory tools (27.08%) to rapidly stimulate production capacity and stabilize public expectations. At the provincial level, while actively responding to central mobilization with motivation tools (39.14%), the use of commands and regulations (27.94%) was significantly higher than at the central level (19.79%), reflecting the urgent need to translate central strategies into concrete local tactics and binding directives.

In the remission stage, the central government shifted its role to that of constructing a unified national supply chain order, with its reliance on commands and regulations peaking at 40.00%, aimed at establishing standardized operational procedures. Complementarily, provincial governments faced peak supply pressures, with motivation tools remaining high at 39.08%, indicating the continued use of financial incentives to sustain local production capacity and circulation efficiency in support of the central framework.

**Fig 12:**
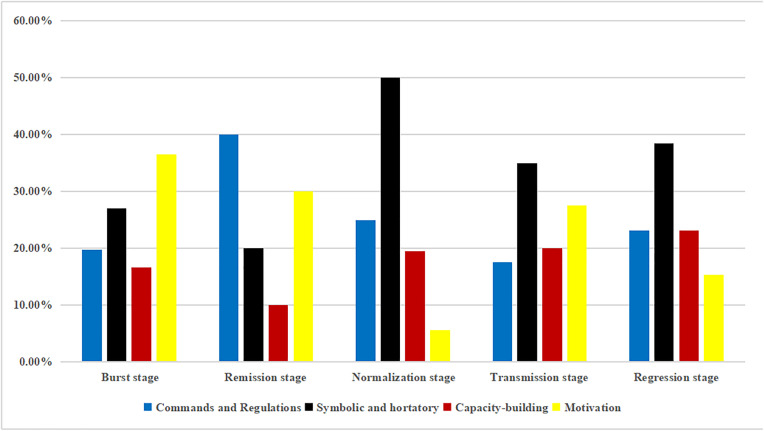
Percentage of policy tools used at COVID-19 development stages at the national level.

**Fig 13:**
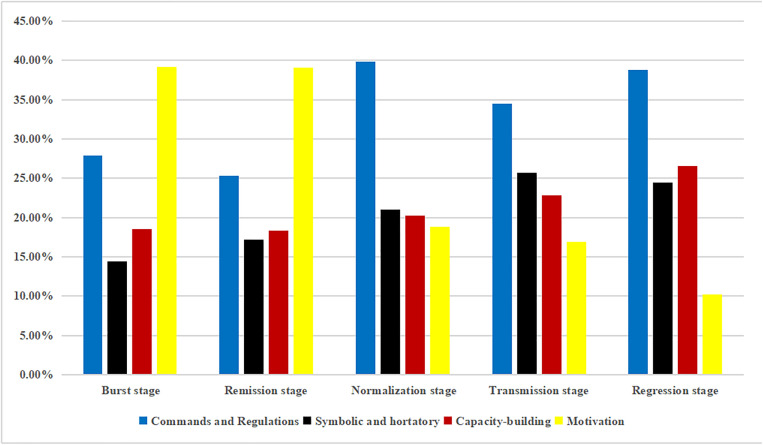
Percentage of policy tools used at COVID-19 development stages at the provincial level.

By the normalization stage, policy priorities shifted markedly. The central government substantially reduced direct intervention, with symbolic and hortatory tools increasing to 50.00%, signaling a transition from “frontline command” to “backline guidance,” emphasizing confidence-building and long-term coordination. In parallel, provincial governments maintained a higher reliance on commands and regulations (39.86% in normalization and 38.78% in regression) alongside a steady rise in capacity-building tools, underscoring that the responsibility for concrete regulatory enforcement and long-term resilience building had been fully delegated to local authorities. This reflects a governance pattern in which the central government “sets the overall direction,” while provincial governments “ensure sustained implementation.” (Figs 12 and 13).

### 3.7. Multidimensional analysis of policy tools, drug supply chain, and developmental stages

National-level policies dynamically adjusted with the developmental stages of the pandemic, shifting from a multi-pronged emergency response to a more macro-oriented governance in the normalization stage. During the burst stage, policies were primarily concentrated in the manufacturing and distribution stages of the supply chain, with symbolic and hortatory tools (10, 16) and motivation tools (15, 20) dominating, supplemented by commands and regulations (9, 13) and capacity-building measures (7, 4). This reflects the government's reliance on multiple tools in parallel to expand production capacity and ensure smooth distribution. R&D received only limited capacity-building support, while the use stage was addressed mainly through symbolic and hortatory tools, and raw material supply was scarcely involved.

In the remission stage, policies were more clearly concentrated in distribution, where symbolic and hortatory tools (15) were most prominent, complemented by commands and regulations (5) and capacity-building (5). R&D was supported mainly through symbolic and hortatory measures (3), while raw material supply, manufacturing, and use were minimally addressed.

During the normalization stage, the focus on distribution persisted, with widespread application of symbolic and hortatory (11) and motivation (7) tools. R&D and manufacturing received sporadic support, while the use of commands and regulations further declined.

In the transmission stage, the policy mix became more diversified. Both R&D and distribution saw the emergence of limited commands and regulations and motivation measures, indicating a transition toward more institutionalized and long-term arrangements.

**Fig 14:**
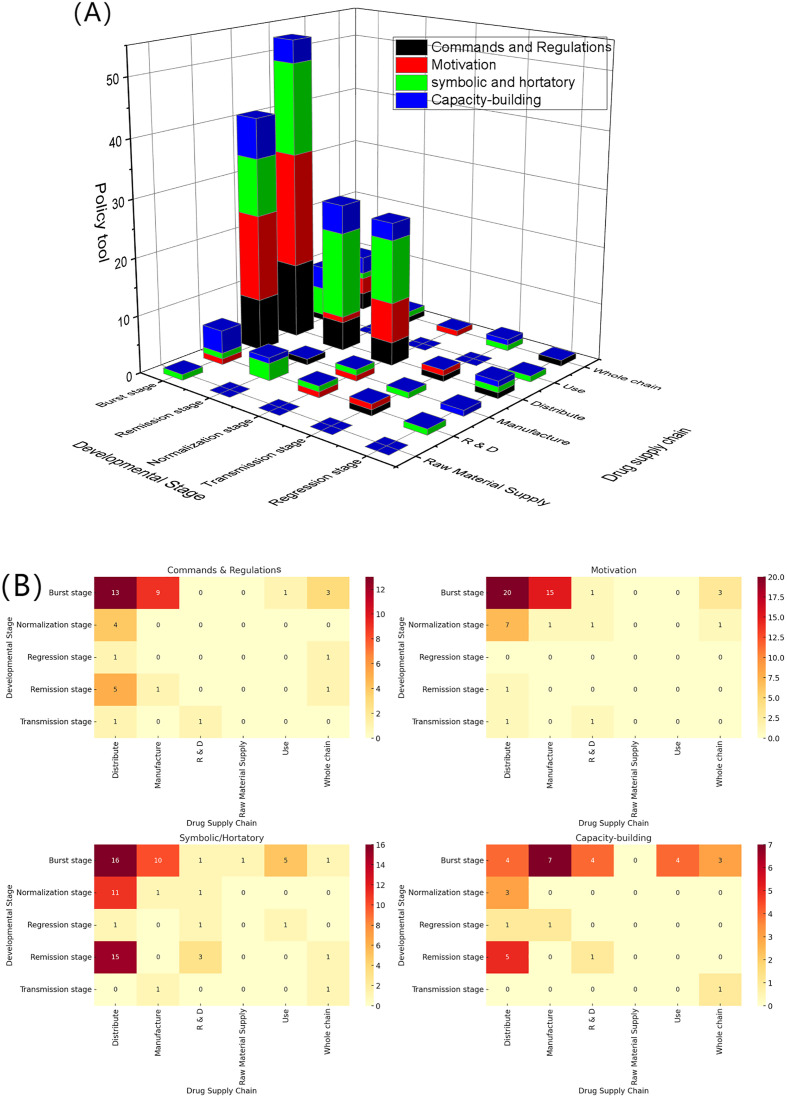
National-level policy tools across developmental stages and supply chains. (A) 3D stacked bar chart. (B) Heatmap.

By the regression stage, the overall intensity of policy intervention declined. Policies were scattered across R&D, manufacturing, distribution, and use, dominated by symbolic and hortatory and capacity-building tools. Commands and regulations appeared only in distribution, suggesting that policy-making was gradually shifting from emergency intervention to routine regulation. The 3D stacked bar chart (Fig 14A) intuitively presents the combinatorial characteristics of policy tools across developmental stages and supply chain segments, while the heatmap (Fig 14B) further quantifies the distribution density of each tool in the stage-segment intersection, both of which provide visual evidence for the above analysis of stage-specific features.

Provincial-level policies initially relied on rigid commands to exert comprehensive control and later shifted toward long-term capacity building. In the burst and remission stages, policies concentrated on manufacturing and distribution, where commands and regulations overwhelmingly dominated. In manufacturing, there were 85 command-and-regulation measures and 47 motivation measures, while in distribution, the figures rose to 111 and 40, respectively. This policy mix highlights the priority of local governments in the early stage of the pandemic: ensuring short-term supply through a combination of administrative mandates and fiscal incentives to rapidly expand production and distribution capacity.

By the regression stage, distribution remained the key focus, with 20 command-and-regulation measures maintaining a high share, while motivation measures dropped sharply to only 3. This shift indicates a transition from a dual emphasis on “motivations and enforcement” to a stronger focus on “maintaining distribution order.” At the same time, capacity-building measures began to appear in R&D (3) and use (1), which, though limited in number, signaled a meaningful turn: provincial governments were starting to consider longer-term support structures and technology deployment.

**Fig 15:**
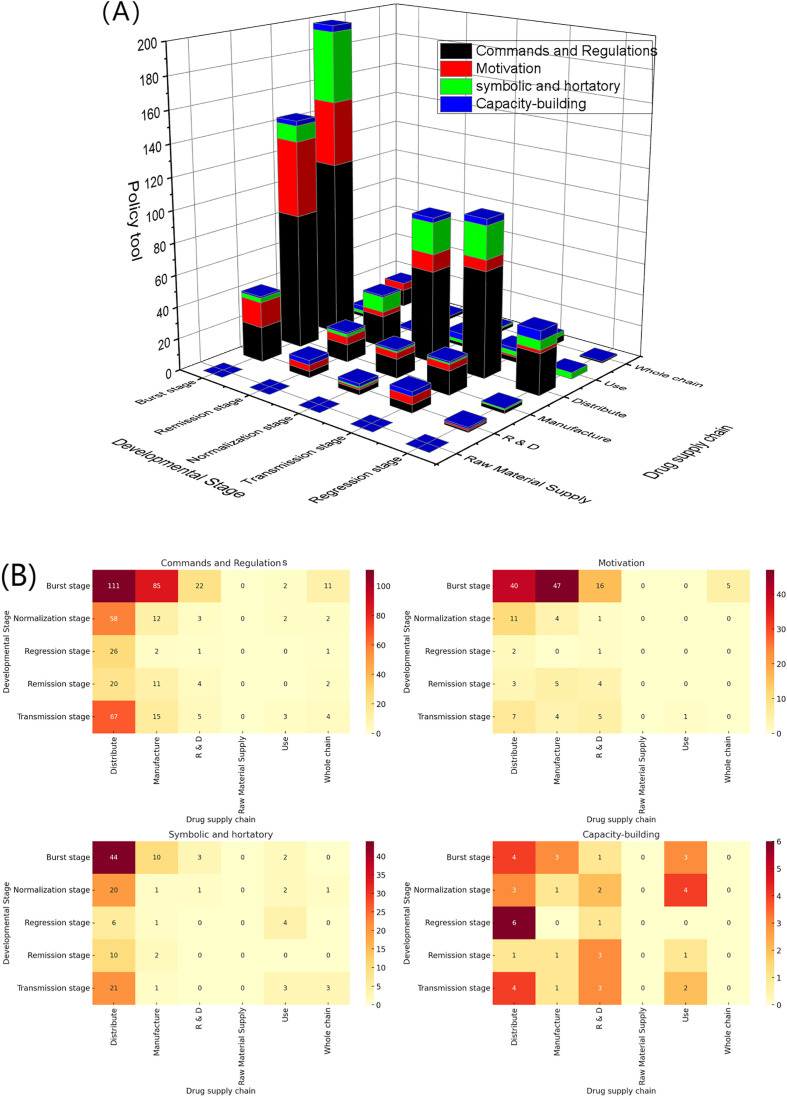
Provincial-level policy tools across developmental stages and supply chains.(A) 3D stacked bar chart. (B) Heatmap.

From the normalization stage through the regression stage, policy resources became increasingly concentrated in distribution. A key change occurred in the regression stage, where capacity-building tools (6) in distribution rose significantly, forming an important complement to commands and regulations (26). This marked a shift in provincial policy priorities from “emergency control” toward “long-term regulation and resilience building,” consolidating emergency experience and laying the foundation for a more robust local drug supply system. In addition, the use stage gained attention during the normalization period, especially through capacity-building (4) and symbolic and hortatory (2) tools, reflecting an extension of policy focus to the end of the supply chain, with greater emphasis on rational drug use and public guidance. These patterns are visually supported by Fig 15A and 15B, which together illustrate the stage-specific distribution and intensity of policy tools.

Overall, national and provincial policies demonstrated complementary functions within the emergency support system, each with distinct emphases. At the national level, policies focused on macro-level planning and stage-specific regulation. The use of policy tools dynamically shifted with the pandemic’ s progression: in the burst stage, motivation and symbolic and hortatory tools were employed to mobilize the nation; in the remission stage, commands and regulations were applied to establish unified order; and in the later stages, the emphasis rapidly shifted to symbolic and hortatory tools, reflecting a strategic transition from direct intervention to macro-level coordination. At the provincial level, policies prioritized rigid enforcement and long-term sustainability. Across all stages, provinces relied heavily on commands and regulations to ensure effective policy implementation. As the pandemic unfolded, the focus of provincial policies gradually shifted from comprehensive early emergency measures to strengthening capacity building in core areas such as distribution in later stages, thereby assuming the primary responsibility for developing localized and sustainable supply systems.

## 4. Discussion

### 4.1. Strengthening the complementarity and connection between national and provincial policies

During the policy-release process, reducing overlap and clarifying the division of responsibilities among all parties are important guarantees for accelerating policy implementation [19]. According to the analysis of policy texts, China’s national and provincial policies in drug emergency supply security have formed a basic division of labor framework of macro-level guidance and local implementation, which played a key role during the pandemic. Building on this existing division of labor, future policy design may benefit from further strengthening coordination between national and provincial authorities to ensure smoother translation of strategic guidance into concrete implementation actions. Provincial government bodies may consider developing more detailed implementation mechanisms corresponding to the macro-level frameworks, reducing repeated efforts and improving contextual applicability. Enhancing communication and information exchange between national and provincial authorities—such as through shared platforms or periodic coordination mechanisms—may also help ensure timely alignment during policy refinement and implementation.

### 4.2. Strengthening coordination and contact among agencies issuing policies

When analyzing the characteristics of institutions issuing policies, the proportion of documents issued jointly by different institutions remains modest, and current coordination and linkage can be further deepened, which may constrain giving full play to the unique advantages of various departments. The existing network diagram shows that inter-agency linkages are already in place and reflect a certain level of collaborative interaction. At the same time, there is scope to further enhance the tightness and depth of collaboration, and to advance the closeness and integration of cooperation among relevant authorities. In this regard, when formulating policies at the national and provincial levels, the working characteristics of each department should be fully considered and the overall planning should be divided. For example, by optimizing the tripartite cooperation mechanism among the drug regulatory, medical insurance, and transportation authorities, the drug regulatory authority feeds back the circulation of the drug market to the medical insurance authority, adjusts the corresponding medical insurance payment catalogue, coordinates the distribution of materials and transport forces in conjunction with the transportation authority according to the needs of all regions, optimizes resource allocation, and ensures timely drug supply. Through the joint issuance of documents, all subjects can strengthen their coordination and cooperation with each other and enhance the fluency of policy implementation.

### 4.3. Optimizing the internal structure of policy tools and improving the balance of tool application

Each policy tool has its own attributes and conditions of use. Collocation to create a complementary effect and coupling balance between different policy tools is essential to ensure the safe and orderly supply of drugs in public health emergencies. During the pandemic, policymaking institutions in China have balanced the use of policy tools, but the internal structure of each policy tool remains to be optimized.

First, in the category of Commands and regulations, the use of “shortage monitoring” measures isrelatively limited. Coding results show that such tools are absent at the national level, and althoughthey appear at the provincial level, they are scattered across individual clauses and have not yetformed a systematic or regular monitoring mechanism. For example, Guangdong Provincerequired procurement platforms to strengthen monitoring of medical supply procurement,incorporate all epidemic prevention-related drugs and consumables into emergencymonitoring, analyze transaction data, establish ledgers for urgently needed items in shortsupply, and dynamically track the supply-demand balance across municipal medicalinstitutions [20]. This indicates that some local governments have begun to experiment with datamonitoring to improve supply chain visibility. In contrast, during the COVID-19 outbreak, the US Food and Drug Administration (FDA) implemented a series of combined approaches to actively monitor national drug supplies and take actions to mitigate or prevent shortages, including issuing guidelines, monitoring product shipments, using discontinuation reporting systems, and maintaining contact with suppliers [21,22]. In the future, China may consider further integrating data from all links of the supply chain on the basis of the existing scheduling and reporting systems, and building a normalized and electronic monitoring system that covers the entire process of production, circulation, and use. This will help realize the transformation from passive response to active early warning, and constitutes a key consideration for enhancing the foresight of emergency response.

Second, within Capacity-building tools, measures related to “Human Resources” remainunderutilized. Coding results show that such policies were not presented as an independent agendaat the national level and, although present at the provincial level, were often embedded withinclauses related to resumption of work and production or the safeguarding of essential supplies. Forexample, Jilin Province emphasized “ensuring labor supply for key enterprises,” organizedonline recruitment through public employment service platforms, and monitored labordemand dynamics for epidemic prevention material manufacturers to forecast employmentneeds [23]. These initiatives reflect that local governments have recognized the importance of humanresources for supply chain stability and have sought to ease localized labor shortages throughflexible deployment and service support. In response to the long-term impact of the coronavirus pandemic on the world labor market, Europe, the United States, Japan, South Korea, and ASEAN countries began opened their borders, lowering the threshold, and adjusting their immigration and employment policies [24]. The Chinese government should also pay more attention to the shortage of the health labor force caused by public health emergencies and formulate policies to supplement the human resources of various subjects in the supply chain.

Third, in the category of symbolic and hortatory, the use of “public opinion and publicity” tools hasbeen adopted to some extent but remains limited overall. At the beginning of a public health emergency, the public will focus on the incident itself, have charged discussion that causes mass panic, and derive new public opinions with the progress of emergencies and the stimulation of various external factors that are often difficult to detect and estimate during an outbreak [25]. Therefore, in response to public health emergencies, greater attention shouldbe paid to the potential role of these tools in stabilizing social expectations and enhancing rationalpublic perception, making them a key direction for improving the overall effectiveness ofemergency communication.

Fourth, motivation policy tool use, China primarily relies on fast track, financial incentives, and other positive incentives, while punitive or disciplinary measures are rare inthe coding results. The US FDA and the Federal Trade Commission (FTC) issued warning letters in March 2020 to seven companies accusing them of selling fraudulent COVID-19 products [26]. in China’s current policy system, motivational tools are more focused onguidance and support; however, future policy design could benefit from combining positive incentives with appropriate constraints to further enhance the standardization andeffectiveness of policy implementation.

### 4.4. Enhancing the synergistic effect of the drug supply chain, developmental stages of public health emergencies, and policy tools

Policy tools are a basic way to achieve policy objectives. The choice of policy tools should match the requirements of each developmental stage for COVID-19 and each process of the supply chain to provide a systematic policy guarantee for the entire process of emergency management. The three dimensions of the developmental stage of COVID-19, each link of the supply chain, and policy tools should be considered to realize scientific and accurate decision-making in emergency management to promote the coupling of policy tools with emergency management practices and increase their effectiveness of policy tools.

During the burst stage, the domestic situation was once chaotic, and public health and safety were under severe threat [27]. At this point, the government needed to strongly intervene in drug supply, stabilize public sentiment, regulate drug purchasing behavior, and focus on scientific research to accelerate R&D progress, thereby providing technological support for epidemic control. Therefore, the burst stage was more suited to the use of Commands and regulations and Capacity-building policy tools: not only issuing compulsory measures to ensure the stability of pharmaceutical logistics and distribution but also targeting the manufacturing process by requiring enterprises to resume production, expand capacity, and adopt technical guidance and financial support to accelerate output expansion. Given the long-term shortage of COVID-19 treatment drugs, the government also needed to strengthen drug R&D capacity-building and supplement it with Motivation tools to ensure faster market entry of R&D outcomes and increase the motivations of R&D entities [28].

During the remission and normalization stages, epidemic control achieved phased success, gradually shifting into an orderly resumption of work and production [29]. In this context, it is important to prioritize the use of symbolic and hortatory, and Capacity-building tools: on the one hand, guiding public opinion and mobilizing society to stabilize expectations; on the other, continuing to strengthen pharmaceutical and medical device R&D, advancing research systems and platform construction, and consolidating the achievements of epidemic prevention and control.

During the transmission stage, variants such as Omicron spread extremely rapidly and demonstrated strong immune escape [30], leading to a short-term surge in drug demand and temporary shortages of analgesics and cold medicines. At this stage, the government not only needed to continue applying Commands and regulations to strictly manage drug production, distribution, and purchasing behavior but also to strengthen Capacity-building tools: improving the quality and accuracy of nucleic acid testing, accelerating vaccine R&D and vaccination progress, and supporting enterprises in increasing the production capacity of special medicines.

During the regression stage, as infection indices gradually declined and multiple COVID-19 treatments were approved for market entry [31], the supply–demand imbalance of medicines eased. At this point, while continuing to use symbolic and hortatory tools to stabilize supply order, it is important to consider expanding the use of Motivation tools: introducing preferential policies to support industrial recovery and long-term development, thereby ensuring the stability and resilience of drug supply.

In summary, the use of various types of policy tools should be linked to the developmental stage of public health emergencies, and future policy design may benefit from issuing targeted policies and improving long-term strategic plans to maximize the supply of drugs and ensure the life safety, health, and well-being of the population, while considering the interests of drug-related parties.

## 5. Limitations and future directions

This study has several limitations. Despite conducting two rounds of screening and coding to comprehensively collect policy documents at the national and provincial levels, some policy documents, particularly specific implementation details and temporary policies at the local level, may not have been included, which could affect the comprehensiveness of the results. Additionally, the study primarily used content analysis and text coding methods, which, while effective for quantifying the use of policy tools, are limited in evaluating the implementation effects of policies and lack an in-depth analysis of their specific impacts during actual execution, Moreover, text analysis itself has inherent limitations, as it may overlook gaps in policy implementation and deviations in practice. For example, the same policy provisions may be implemented with significantly different effects across regions and institutions, which is difficult to capture solely through text-based analysis.

Subjectivity was involved in the classification and coding of policy tools, and differences in understanding among researchers could affect the consistency of the results. The study focused on static analysis of policy texts and did not adequately consider the dynamic changes and practical adjustments in policy implementation across different times and regions. Finally, the study concentrated on policies themselves and did not fully account for external factors such as international aid and socio-economic conditions, which could significantly influence actual policy implementation.

Future research could be expanded in three directions: first, extending the scope of analysis to municipal and grassroots-level policies to capture more operational measures; second, incorporating field interviews, case studies, or surveys to validate the gap between policy design and actual implementation outcomes; and third, strengthening comparative studies between China and other countries to identify policy optimization pathways tailored to the Chinese context.

The explosive and widespread characteristics of public health emergencies bring various challenges to the supply of drugs and require strong government intervention to stabilize the drug market. It can be seen from this study that at different stages of public health emergencies, relevant government departments use various policy tools to comprehensively cover all aspects of the drug supply chain and strive to enhance the stability of drug supply. At the same time, there is room for improvement in the coordination of government subjects and the balance of policy tools. This study proposes countermeasures and suggestions to improve China's ability to manage drug supplies during public health emergencies to protect people's lives and health [32].

## Supporting information

S1 Table Centrality measures of the national-level joint policy-issuing agency network. (PDF)

S2 Table Centrality measures of the provincial-level joint policy-issuing agency network. (PDF)

S1 File Dataset. (XLSX)

## Abbreviations

FTC: Federal Trade Commission
FDA: Food and Drug Administration
