# Supplementary material for: The practice of drug emergency supply in China during the COVID-19 pandemic: A policy mix perspective
Source: PLoS One. 2025 Dec 30;20(12):e0337700. doi: 10.1371/journal.pone.0337700 (PMC12753071; doi:10.1371/journal.pone.0337700)
Supplement: S1 Table — (PDF) [file pone.0337700.s001.pdf]

S1 Table. Centrality Measures of the National-Level Joint Policy-Issuing Agency Network

No.	Agency	Centrality Measures			
		Degree	Closenes	Eigenvec	Between
1	Central Rural Work Leadership Group	1.0000	137.0000	1.0000	0.0000
2	China Banking and Insurance Regulatory Commission	16.0000	60.0000	0.0000	8.4050
3	China National Intellectual Property Administration	15.0000	65.0000	0.0000	0.0000
4	China Securities Regulatory Commission	4.0000	74.0000	0.0000	0.0000
5	China State Railway Group Co., Ltd.	5.0000	72.0000	0.0000	0.0000
6	Civil Aviation Administration of China	5.0000	72.0000	0.0000	0.0000
7	General Administration of Customs	21.0000	61.0000	0.0000	3.5740
8	Ministry of Agriculture and Rural Affairs	3.0000	83.0000	0.0000	0.0000
9	Ministry of Civil Affairs	16.0000	60.0000	0.0000	15.0060
10	Ministry of Commerce	19.0000	64.0000	0.0000	0.7500
11	Ministry of Culture and Tourism	13.0000	65.0000	0.0000	0.0000
12	Ministry of Education	12.0000	62.0000	0.0000	0.0000
13	Ministry of Finance	38.0000	54.0000	0.0000	55.6660
14	Ministry of Housing and Urban-Rural Development	12.0000	62.0000	0.0000	0.0000
15	Ministry of Human Resources and Social Security	14.0000	62.0000	0.0000	0.0000
16	Ministry of Industry and Information Technology	33.0000	60.0000	0.0000	8.7240
17	Ministry of Public Security	24.0000	56.0000	0.0000	24.3300
18	Ministry of Science and Technology	12.0000	62.0000	0.0000	0.0000
19	Ministry of Transport	6.0000	68.0000	0.0000	2.1170
20	National Administration of Disease Prevention and Control	6.0000	76.0000	0.0000	1.7490
21	National Administration of Traditional Chinese Medicine	2.0000	84.0000	0.0000	0.0000
22	National Audit Office	8.0000	74.0000	0.0000	0.0000
23	National Development and Reform Commission	33.0000	56.0000	0.0000	15.0500
24	National Forestry and Grassland Administration	13.0000	65.0000	0.0000	0.0000
25	National Health Commission	29.0000	53.0000	0.0000	98.2120
26	National Healthcare Security Administration	5.0000	77.0000	0.0000	2.0000
27	National Medical Products Administration	26.0000	56.0000	0.0000	24.3300
28	Office of the Central Cyberspace Affairs Commission	13.0000	65.0000	0.0000	0.0000
29	People's Bank of China	37.0000	48.0000	0.0000	71.2230
30	Publicity Department of the Communist Party of China Central Committee	13.0000	65.0000	0.0000	0.0000
31	State Administration for Market Regulation	33.0000	51.0000	0.0000	31.9320
32	State Administration of Foreign Exchange	4.0000	74.0000	0.0000	0.0000
33	State Council of the People's Republic of China	1.0000	137.0000	1.0000	0.0000
34	State Post Bureau	13.0000	65.0000	0.0000	0.0000
35	State Taxation Administration	27.0000	51.0000	0.0000	31.9320
36	State-owned Assets Supervision and Administration Commission of the State Council	12.0000	62.0000	0.0000	0.0000
