# Supplementary material for: The practice of drug emergency supply in China during the COVID-19 pandemic: A policy mix perspective
Source: PLoS One. 2025 Dec 30;20(12):e0337700. doi: 10.1371/journal.pone.0337700 (PMC12753071; doi:10.1371/journal.pone.0337700)
Supplement: S2 Table — (PDF) [file pone.0337700.s002.pdf]

S2 Table. Centrality Measures of the Provincial-Level Joint Policy-Issuing Agency Network.

No.	Agency	Centrality Measures			
		Degree	Closenes	Eigenvec	Between
1	Agriculture and Rural Affairs Authorities	21.0000	66.5000	0.0725	21.0390
2	Audit Bureau	30.0000	71.5000	0.2010	0.6610
3	Big Data Center	11.0000	70.5000	0.0660	0.6670
4	Civil Defense Office	13.0000	73.0000	0.0310	0.5220
5	Commerce Bureau	69.0000	57.0000	0.3795	82.9920
6	Customs	14.0000	69.5000	0.0865	0.4540
7	Department of Civil Affairs	2.0000	104.5000	0.0130	0.0670
8	Department of Housing and Urban-Rural Development	10.0000	79.5000	0.0260	1.9470
9	Department of Human Resources and Social Security	18.0000	70.0000	0.1020	0.1430
10	Department of Natural Resources	10.0000	79.5000	0.0260	1.9470
11	Development and Reform Authorities	104.0000	54.5000	0.6000	101.2640
12	Ecology and Environment Bureau	13.0000	73.0000	0.0310	0.5220
13	Industry and Information Technology Authorities	91.0000	57.0000	0.5135	94.9140
14	Emergency Management Bureau	10.0000	87.0000	0.0230	0.0000
15	Finance authorities	140.0000	57.0000	0.8395	44.2190
16	Financial Supervision and Administration	32.0000	70.5000	0.1320	19.3730
17	Fire and Rescue Corps	10.0000	79.5000	0.0260	1.9470
18	Forestry Bureau	4.0000	82.0000	0.0150	0.0000
19	Intellectual Property Office	3.0000	80.5000	0.0195	0.0000
20	Justice Bureau	13.0000	73.0000	0.0310	0.5220
21	Local CBIRC Office	35.0000	65.5000	0.1220	33.3920
22	Local CSRC Office	9.0000	84.5000	0.0270	0.2500
23	People’s Bank of China (local branches)	51.0000	66.0000	0.2740	14.1390
24	Market Supervision Authorities	108.0000	48.5000	0.5110	353.7970
25	Narcotics Control Commission Office	10.0000	78.5000	0.0370	19.4750
26	Provincial Administration of Traditional Chinese Medicine	15.0000	74.5000	0.1285	0.6670
27	Provincial Health Commission	60.0000	67.5000	0.5460	50.6350
28	Provincial Healthcare Security Administration	80.0000	60.5000	0.9550	120.5150
29	Provincial Medical Products Administration	92.0000	57.5000	0.5465	137.8200
30	People's Government General Office	4.0000	131.0000	0.0090	0.0000
31	Pharmaceutical Commerce Association	2.0000	92.0000	0.0145	0.0000
32	Public Security Authorities	48.0000	58.5000	0.2390	82.4840
33	Road Transport Administration	11.0000	76.5000	0.0200	21.9820
34	Science and Technology Commission	15.0000	74.0000	0.0935	15.0340
35	Science and Technology Park Administrative Committee	13.0000	75.5000	0.1120	0.4670
36	State-owned Assets Supervision and Administration Commission of the State Council	15.0000	70.0000	0.0880	5.6080
37	Taxation Bureau	18.0000	70.0000	0.1075	0.5330
